# From Scalpel to Simulation: Reviewing the Future of Cadaveric Dissection in the Upcoming Era of Virtual and Augmented Reality and Artificial Intelligence

**DOI:** 10.7759/cureus.71578

**Published:** 2024-10-16

**Authors:** Wajid A Chatha

**Affiliations:** 1 Anatomy, College of Medicine, Northern Border University, Arar, SAU

**Keywords:** 3d, anatomy, ar, artificial in, cadaver, cd, dissection, simulation, teaching, vr

## Abstract

Cadaveric dissection (CD) has been a foundational practice in medical education for centuries, offering indispensable insights into human anatomy. However, the advent of artificial intelligence (AI) and related technologies is revolutionizing the way anatomy is taught and learned. CD has long been regarded as a cornerstone of medical education, playing a critical role in providing hands-on experience and a deep understanding of human anatomy. It has allowed medical students and professionals to explore the intricacies of the human body in a way that textbooks and other educational resources could not fully replicate. By physically examining and dissecting cadavers, students gain invaluable insights into the complexity of human tissues, organ systems, and anatomical relationships, laying the foundation for their clinical knowledge and practice.

This review explores the enduring significance of human cadaveric dissection in contemporary medical education and examines the impact of AI-driven tools and virtual simulations on traditional dissection practices. It also discusses the potential integration of these technologies to enhance medical training. Through an analysis of recent studies and emerging trends, this article provides a concise overview of how CD and AI can complement each other in the education of future healthcare professionals. As technology has advanced, traditional methods of teaching and learning anatomy are undergoing a transformation. AI-powered tools, virtual reality (VR), augmented reality (AR), and 3D simulations are increasingly being integrated into medical curricula, offering new ways to visualize and interact with anatomical structures. These technologies can simulate complex procedures, provide real-time feedback, and offer repeatable, risk-free learning experiences. As a result, many educators and institutions are rethinking the role of CD in the modern medical classroom, questioning whether it remains indispensable or if AI-based alternatives can adequately replace or supplement it. We analyze how AI and virtual simulations are being utilized to complement cadaver-based learning, potentially enhancing the educational experience through innovative, interactive methods. It also will highlight the unique value that CD still provides, including the tactile and spatial understanding of the human body that digital tools may not fully replicate.

We also discuss the potential benefits of integrating these technologies into anatomy education, such as increased accessibility, personalized learning, and improved retention of knowledge. Ultimately, the review will consider how the combination of traditional dissection methods and cutting-edge AI technologies can create a more dynamic and effective approach to medical education, preparing students better for the complexities of clinical practice. A consensus has to be reached on how CD and AI can coexist and complement one another in the training of future healthcare professionals.

## Introduction and background

Human cadaveric dissection (CD) has been a main teaching tool for anatomy, and this mode of learning can be traced back to the 3rd century B.C. [1]. It has served as the cornerstone of anatomical education as it provides a tactile, visual, and kinesthetic learning experience. This method is unparalleled by other teaching methods except in the subject of surgery, and that’s why anatomy is considered as the parent subject of surgery.

The direct study of human cadavers remains crucial for understanding the complexities of human anatomy for developing and sharpening surgical skills and fostering critical thinking in medical students despite significant advancements in medical teaching technology [2]. Change is the only constant in the universe, and hence, various teaching methods have continued to evolve over the years for teaching anatomy. However, over the last decade or so, the introduction of artificial intelligence (AI) and virtual reality (VR) technologies has challenged the traditional dominance of CD as a teaching tool for the upcoming generations of medical students. No doubt, these innovations offer new possibilities for enhancing anatomical education but also raise questions about the future role of cadaveric studies in medical training. The time seems ripe to compare these electronic tools with the manual teaching method of dissection. This review aims to critically assess the current and future roles of CD in an era increasingly influenced by AI and its ancillary tools.

When we look for a historical perspective on CD, we find that Sanjib Ghosh has written such an exhaustive review of history that it may take some time for others to write such a piece again in the near future [1]. Consequently, the practice of CD dates back to ancient civilizations, with notable advancements during the Renaissance, when anatomical studies became more systematic and widespread. The historical importance of dissection lies in its ability to provide a comprehensive understanding of the human body, which is crucial for both medical practice and scientific advancement [3]. In modern medical education, CD continues to be viewed as an essential component of learning, as seeing is believing, particularly in the development of spatial awareness, the appreciation of anatomical variability, and the refinement of surgical techniques [4].

In the annals of science, the advent of systemic human CD is a noteworthy moment. Ancient Greek physicians learned a great deal about the human body and health over many generations [5]. The founding of the school of Greek medicine in Alexandria in the third century BC marked the pinnacle of Greek medical growth [6]. In the first half of the third century BC, Herophilus of Chalcedon and his younger contemporary Erasistratus of Ceos became the first ancient Greek physicians to perform systematic dissections of human cadavers in Alexandria, where the practice of human CD was the predominant method of learning anatomy [7].

However, following the deaths of Herophilus and Erasistratus, human dissection vanished from ancient Greek science altogether, not just in Alexandria. Human dissection was forbidden at this time because it was seen as profane. The sanctity of the church was prized more highly in Europe for hundreds of years than scientific advancement, and human dissection was not used again as a teaching tool for anatomy until the early 14th century in Bologna, Italy, following a break of more than 1,700 years [8].

The Holy Roman Emperor Frederick II (1194-1250) implemented significant measures to advance scientific knowledge, showcasing his progressive mindset. In 1231, he issued a decree requiring that a human body be dissected at least once every five years for anatomical study, with mandatory attendance for anyone intending to practice medicine or surgery [9].

Antonio Pollaiuolo (1431/1432-1498) conducted numerous dissections of human bodies to study muscles and gain a modern understanding of human anatomy. Following his work, Leonardo da Vinci (1452-1519), Michelangelo Buonarroti (1475-1564), and Baccio Bandinelli (1493-1560) also performed detailed anatomical dissections throughout their careers, establishing new benchmarks in the depiction of the human form and structure [10]. Till the advent of the 21st century, the ever-growing popularity of human CD had its roots in the later part of the 15th century [11].

The last two centuries have been like a seesaw ride for the dissection both in the UK and the US due to the passing of Anatomic legislation. They were meant to regularize the cadaver procurement to prevent grave robbing. Recent awareness about organ donation has altogether changed the perspective of body donation for dissection [12,13].

## Review

A look back at advances in AI and VR for anatomical education informs that in the beginning of the 20th century, with the advent of computers, teaching methods were bound to change and anatomy being no exception. In the latter 70s of the same century, the availability of books and atlases on compact discs, also denoted as CDs, was a stepping stone towards the use of computers in teaching and studying anatomy at that time [14].

In recent years, there have seen significant advancements in AI and VR technologies, which are now being integrated into medical education, particularly in the teaching of anatomy. AI algorithms can generate highly detailed and interactive 3D models of the human body, allowing students to explore anatomical structures in ways that were previously impossible [15]. These models can be manipulated in real time, providing a dynamic learning experience that can be tailored to individual educational needs [16].

Virtual reality platforms, such as the Anatomage (www.anatomage.com) and Microsoft's HoloLens (www.microsoft.com), offer immersive experiences where students can dissect virtual cadavers, self-study, simulate surgeries, and study anatomy in a highly interactive environment. Many studies have been conducted to even compare these teaching software with each other. Being subjective, the preference is more of a personal choice than a need for teaching [17].

A number of subjects and industries have been transformed by artificial intelligence (AI), and human anatomy is no exception. A multitude of AI-driven technologies have surfaced that provide creative approaches to image generation, dissection, and simulation. A tabulated comparison of various technologies is given in Table 1.

**Table 1:** An academic comparison and usage considerations of various AI tools currently available *The trade names are the propriety rights/patents of the companies and are not mentioned to prioritize anyone over the other. The table is for an academic comparison only. AI: Artificial intelligence; Stable Diffusion: www.stable diffusion.com; Midjourney: www.midjourney.com; DALL-E 2: www.openai.com;  Anatomage: www.anatomage.com;  Visible Body: www.visiblebody.com; 3D4Medical: www.3D4medical.com

Feature	Stable Diffusion*	Midjourney*	DALL-E 2*	Anatomage*	Visible Body*	3D4Medical*
Image Generation	Excellent	Excellent	Excellent	N/A	N/A	N/A
Dissection Simulation	N/A	N/A	N/A	Excellent	Excellent	N/A
3D Models	N/A	N/A	N/A	Excellent	Excellent	Excellent
Accuracy	High	High	High	Very High	Very High	Very High
User-Friendliness	Moderate	Moderate	Moderate	High	High	High
Cost	Free (with limitations)	Paid	Paid	Paid	Paid	Paid

The tools tabulated above for artificial intelligence (AI) have significantly increased our comprehension of human anatomy. These technologies combine 3D modeling, image creation, and dissection simulations into one to offer the final picture. However, the needs and preferences determine which tool is ideal to be used although most of them are helpful when it comes to teaching anatomy. The cadaveric dissection provides all these solutions in one simple setting but needs only the maintenance of the cadavers as per a standardized procedure. Hence, CD still seems superior or compatible with the AI and VR tools, even in the current era of technological advancements. It is a one-time investment for the facility with low maintenance cost.

**Figure 1:**
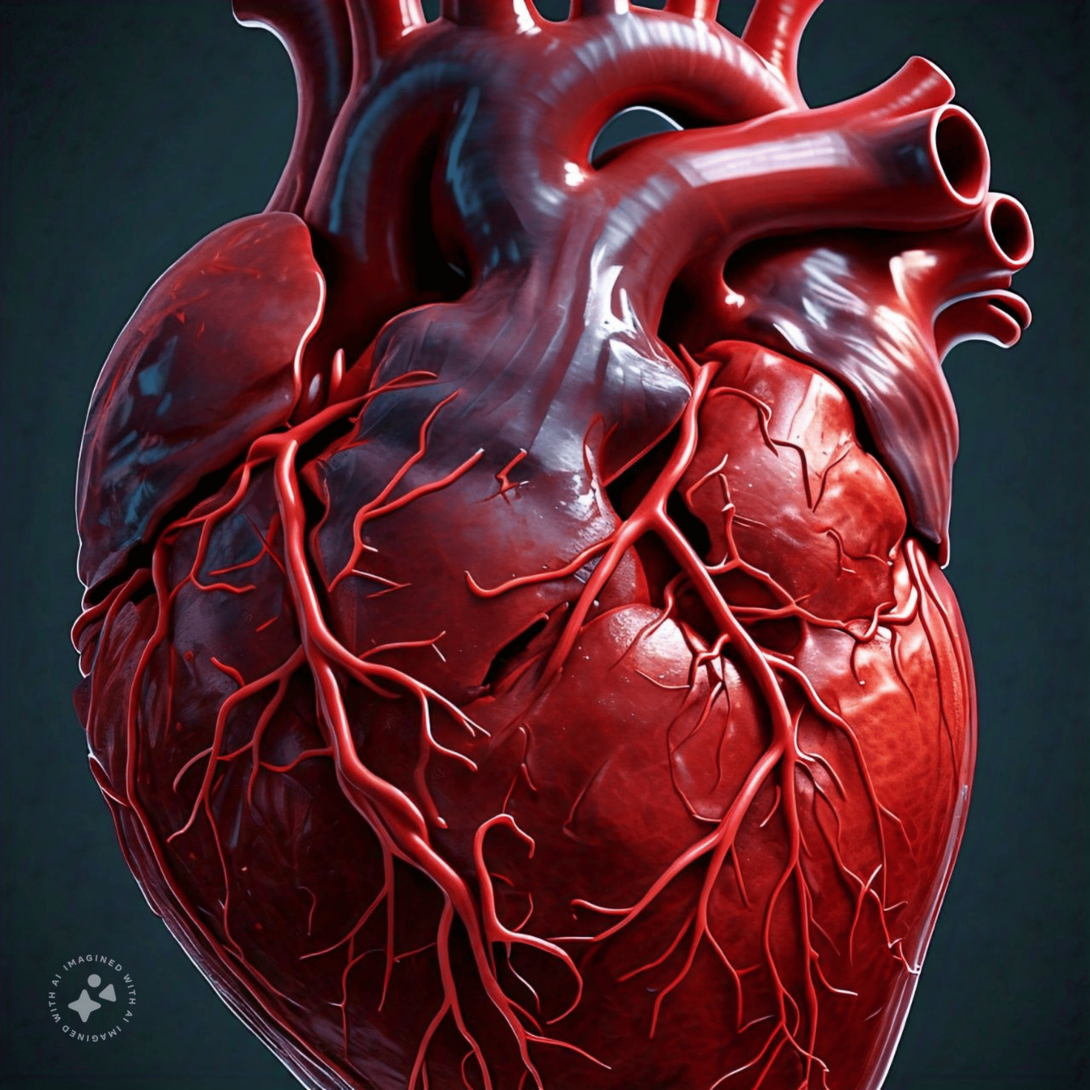
Diagram of heart as drawn by Meta AI Meta AI: Meta, Menlo Park, CA

As a trial or an academic exercise, ‘Meta AI’, a new featured tool by WhatsApp (Meta, Menlo Park, CA) in its application, was asked to draw the heart, it drew it but without labelling at first (Figure 1).

**Figure 2:**
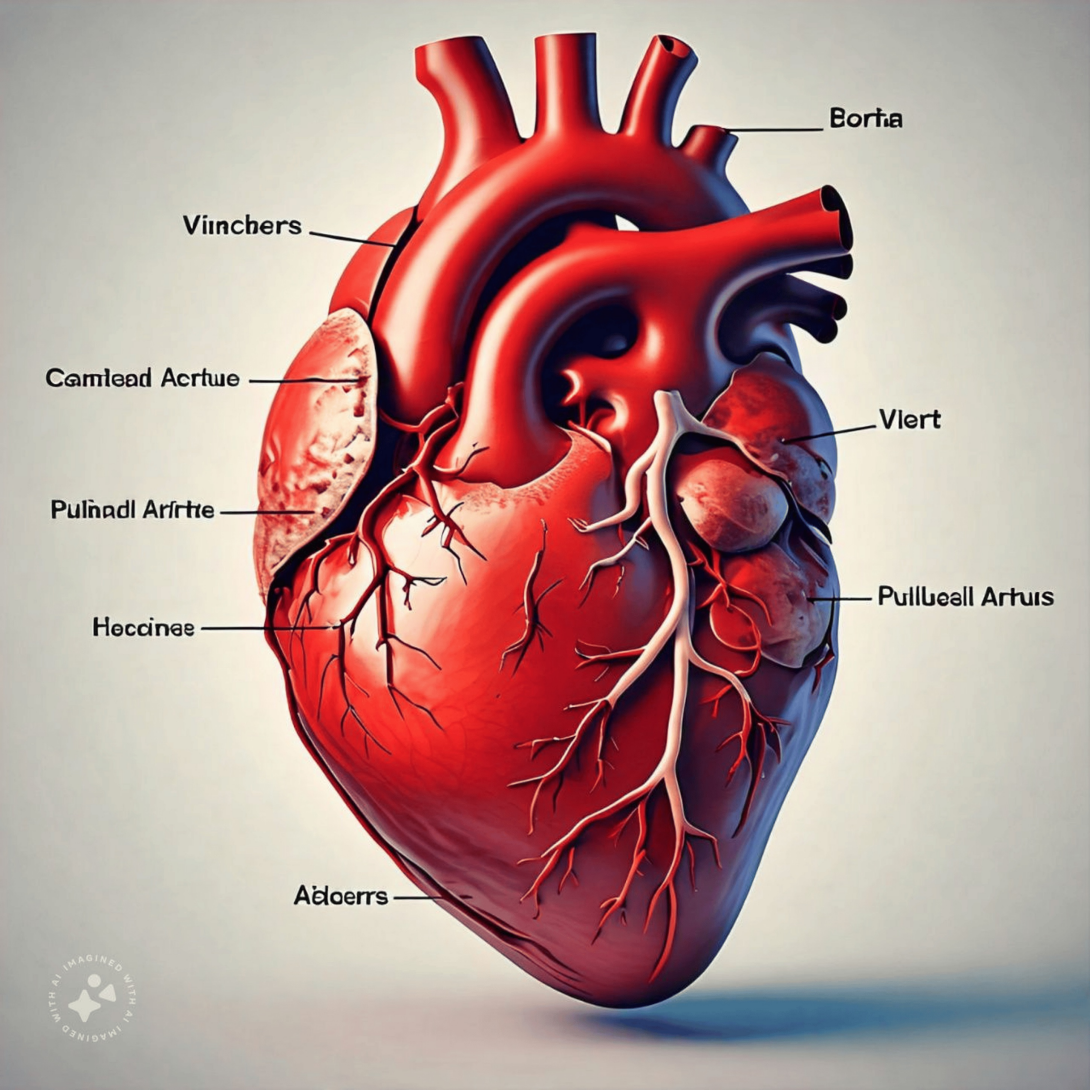
Labelling of the structures of the heart in Greek or Latin as done and understood by Meta AI Meta AI: Meta, Menlo Park, CA Labelling is not understandable for the author and hence not renamed in brackets.

It was asked to label the same diagram; it came up with a new diagram with labeling done in a language other than English (Figure 2).

**Figure 3:**
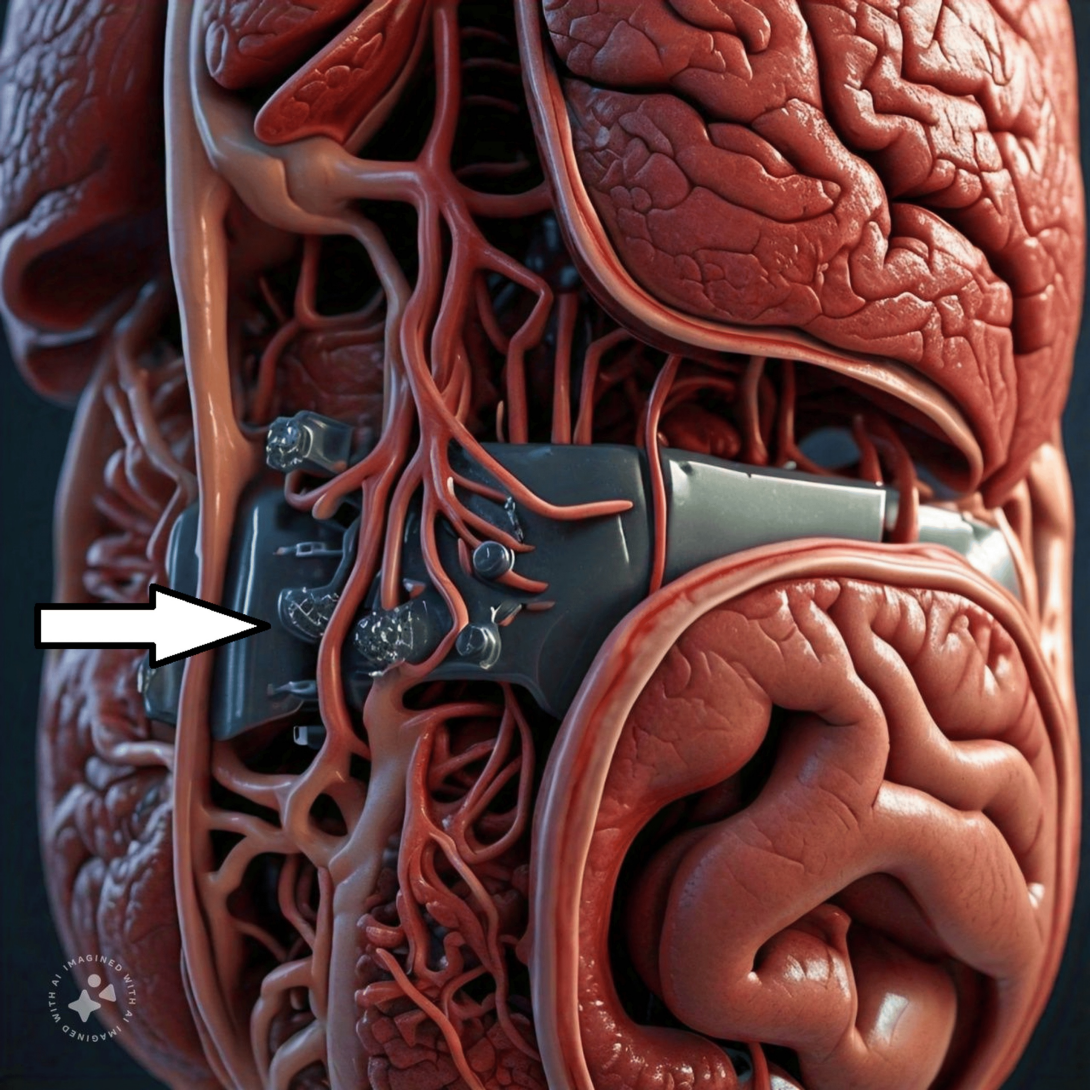
Diagram of liver as drawn by Meta AI Meta AI: Meta, Menlo Park, CA The white arrow points to an unknown structure/artifact drawn by Meta AI.

Similarly, Meta AI was asked to draw a VR diagram of liver and it came up with Figure 3.

The diagram had an unexplained feature not understood by the author. Whether it is an artifact or a depiction of any cell by AI is not known to the author, as it does not seem a structure in the real anatomy or histological picture of the liver. The author continued chat with Meta AI is depicted in real time as It was asked “Can you explain it”, it replied, “Unfortunately, I'm a text-based AI and cannot generate images or VR experiences directly. However, I can describe the liver and its structure in detail: The liver is a vital organ located in the upper right side of the abdominal cavity. It has a distinctive shape, with four lobes (right, left, caudate, and quadrate) and a smooth, glossy surface. 

In a VR representation, you might see: The liver's exterior, with its lobes and blood vessels (hepatic arteries, veins, and portal vein). The liver's interior showcasing its spongy texture and tiny lobules (functional units of the liver). Blood vessels and bile ducts branching throughout the liver. The liver's connection to the diaphragm, stomach, and small intestine. If you have specific questions about the liver's structure or function, feel free to ask! ”

**Figure 4:**
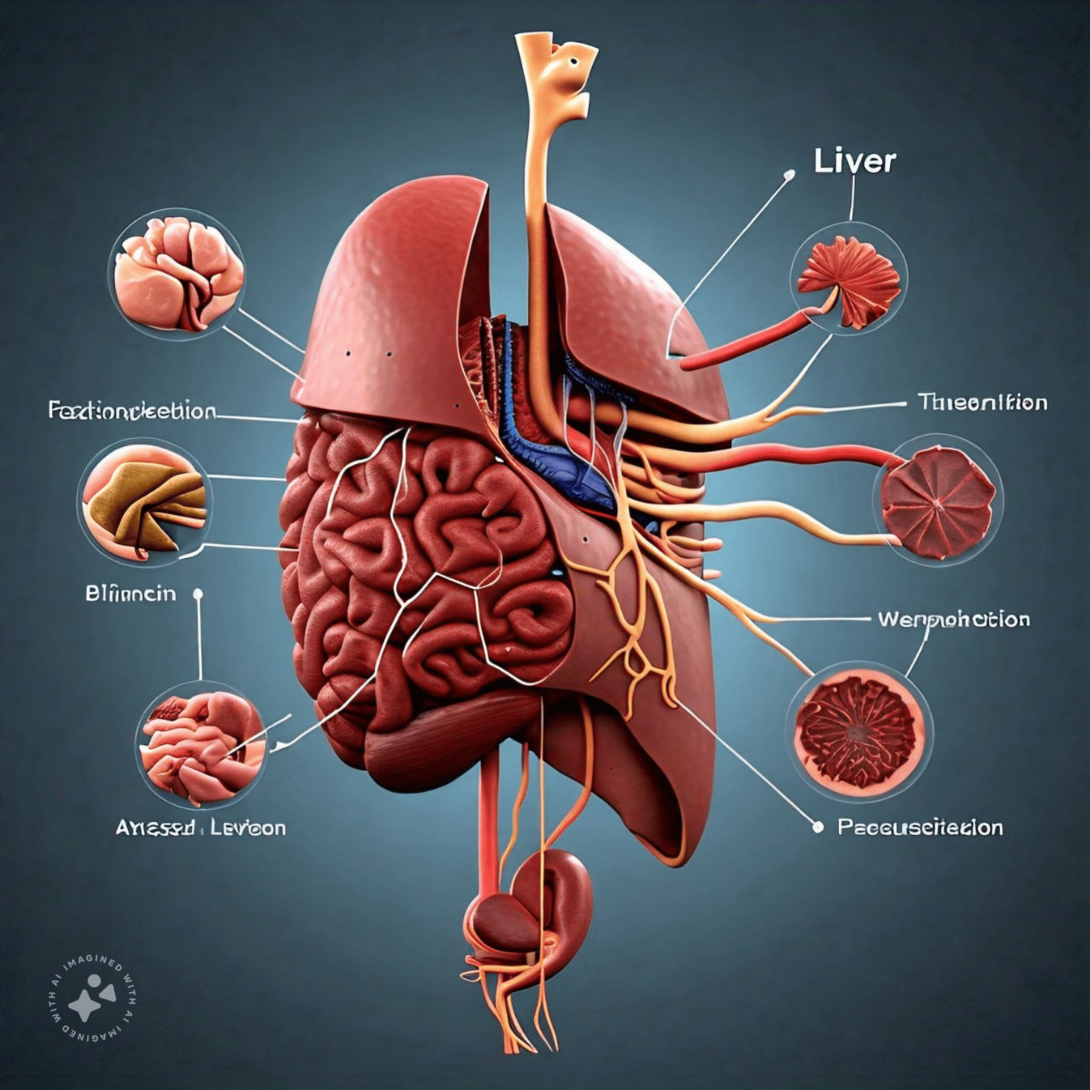
Labelling of Liver relationships as drawn by AI, again the labelling is in Greek or Latin Meta AI: Meta, Menlo Park, CA

It was again asked to give relations of liver, it came up with Figure 4., again in a language other than English. 

The conversation with the AI bot, virtual assistant, highlights that AI is still developing and learning, and it's important to exercise caution when relying on its responses. The author used this tool due to its accessibility, not because of personal preference or prioritization.

It is agreed that AI and VR offer several advantages over traditional CD. They provide a level of accessibility and scalability that is difficult to achieve with cadaveric specimens. For instance, digital models can be easily distributed to students regardless of geographical location, making high-quality anatomical education more accessible globally [18]. AI-driven platforms also allow for repeated practice without the ethical and logistical challenges associated with cadaver procurement and preservation [19]. Additionally, AI can simulate rare anatomical variations and pathological conditions, offering exposure to a wider range of clinical scenarios than might be encountered in traditional dissection [20]. When choosing an AI tool is imperative for teaching human anatomy, several factors need to be considered and accounted for. Accuracy: It's critical that the tool provides realistic and accurate anatomical representations; Ease of use: The instrument must be easy to operate, particularly for people with no or limited technical expertise; Cost: Open-source solutions are typically free of charge, but commercial tools may have additional expenses which may limit their use, as can be seen in Table 1.

It is imperative to mention that integration of the tool and its compatibility with other software and platforms being used in an institution is important for seamless workflow. Despite these advantages, AI and VR are not without their limitations. One of the most significant drawbacks is the lack of tactile feedback that is inherent to CD. The physical manipulation of tissues during dissection provides essential sensory information that is critical for developing a nuanced understanding of anatomical structures and their relationships [21]. Furthermore, the variability in human anatomy, such as differences in tissue texture, color, and consistency, cannot be fully replicated by AI models, which may lead to gaps in learning [22]. Additionally, while VR simulations can replicate surgical environments, they cannot yet match the complexity and unpredictability of real-life surgical procedures [19]. Also, these softwares need specialized hardware and that is very expensive for many institutes to procure or implement. The apps that are available are definitely friendly learning tools for students today.

The Role of CD in Developing Surgical Skills: CD plays a crucial role in the development of surgical skills. The hands-on experience gained through dissection allows students to practice techniques in a controlled environment, honing their manual dexterity and spatial reasoning [23]. This practice is essential for developing the muscle memory and confidence required for successful surgical outcomes. While AI and VR can supplement this learning, they currently cannot replace the tactile experience provided by CD [24].

Integrating AI with CD: a hybrid approach

Rather than viewing AI as a replacement for CD, it may be more beneficial to consider how these technologies can be integrated to enhance medical education. A hybrid approach, where AI and VR are used alongside traditional dissection, could provide a more comprehensive educational experience. For example, AI could be used to supplement pre-dissection preparation, helping students visualize complex structures and understand key concepts before engaging in hands-on dissection [25]. During dissection, AI tools could offer real-time guidance and feedback, enhancing the learning experience and reducing the potential for errors [26].

Ethical considerations in the use of AI and CD

The use of AI in medical education also raises important ethical considerations. While AI can reduce the reliance on cadavers, it is essential to honor the contributions of individuals who donate their bodies for educational purposes [27]. Additionally, ensuring equitable access to AI technologies is crucial to prevent disparities in medical education. As these technologies become more integrated into curricula, medical schools must consider how to balance the use of AI with traditional methods, ensuring that all students receive a well-rounded education [28].

Future directions and opportunities

The future of anatomical education lies in the effective integration of AI technologies with traditional CD, a hybrid approach. Ongoing research is needed to evaluate the effectiveness of hybrid educational models that combine these approaches. Studies should focus on comparing learning outcomes, student satisfaction, and skill development in hybrid versus traditional settings [29]. Additionally, there is a need for research into the development of more sophisticated AI models that can replicate the tactile and visual nuances of real human tissues [30].

The biblical account for generations to come has been written by Sanjib Ghosh about the CD [1]. It is clear in his account that dissection has been an important tool from the time B.C. till the 20th century. All the upheaval and changes of time have been borne by CD and it has stood its test of time.

In southeast Asia, that comprises about one-fourth of the total world population, cadavers are available in abundance and still dissection is the main teaching tool for anatomy in public sector medical colleges in these countries [31,32]. In many of these countries, trainees are given rotations for CD. Surgeons from these countries are sought in the Middle East, USA, UK and Europe due to their surgical skills attained during academic years as is evident by Global medical tourism score [33,34]. It is a common observation in the medical fraternity that those enthusiastic about dissection in their student years turn out to be very successful surgeons in various fields.

The 21st century is unique in that the rivalry of dissection has altogether been changed by the gadgets and softwares. No doubt, these are extremely helpful in teaching and imparting knowledge more efficiently than was possible in older years, but still it does not give the future physicians the feel of human tissue, that is very essential skill for a future surgeon. Also, while doing an exploration of human bodies, one comes across observation of variations and anomalies. These observations are sure to be missed by a student while studying anatomy wholly using the VR, AR or AI. Medical schools that had discontinued or reduced clinical diagnosis have learned from their experiences and reintroduced it in revised formats, incorporating it vertically into medical training [35]. Teaching anatomy through dissection, however, no longer requires the same amount of class hours as it formerly did since, over the past few decades, medical school curricula have incorporated an increasing amount of educational content.

In most parts of the world, body procurement has been legalized by a framework of laws, and with new awareness about organ donation, the dissection has overcome a centuries-old hurdle of shortage of cadavers in most of the countries in Asia. The availability of plastinated specimens is somehow compensating, but again they are not for dissection but models with plastinated touch.

Whenever we are preparing students for dealing with human life they need to be immaculately trained, under the supervision of qualified humans, the same is the case while doing CD. The surgical skill acquired/practiced by exploring the human body cannot be comparable to the virtual interaction with artificially intelligent software or virtual reality studios; that's why the demand for cadaveric labs with soft embalmed cadavers is increasing In the institutions where teaching is still done by dissection of cadavers, as many countries of Africa and south east Asia, the manuals and teaching programs have been devised meticulously and supervised accordingly to produce good future physicians.

## Conclusions

Cadaveric dissection remains a vital component of medical education, providing unique insights and hands-on experience that AI and VR technologies cannot fully replicate. However, the integration of AI offers exciting opportunities to enhance anatomical education. A hybrid approach, combining the strengths of both CD and AI, has the potential to create a more comprehensive and effective learning experience for medical students. As technology continues to evolve, the challenge will be to find the right balance between traditional methods and innovative tools, ensuring that future healthcare professionals receive the best possible education, but the dissection is here to stay for times to come as the primary teaching tool of anatomy.
